# Assessing the effect of urbanization on regional-scale surface water-groundwater interaction and nitrate transport

**DOI:** 10.1038/s41598-022-16134-1

**Published:** 2022-07-22

**Authors:** Bisrat Ayalew Yifru, Il-Moon Chung, Min-Gyu Kim, Sun Woo Chang

**Affiliations:** 1 Department of Water Resources and River Research, Korea Institute of Civil Engineering and Building Technology, Goyang, 10223 Republic of Korea; 2 Civil and Environmental Engineering Department, University of Science and Technology, Daejeon, 34113 Republic of Korea

**Keywords:** Environmental sciences, Hydrology

## Abstract

Identifying regional-scale surface water-groundwater interactions (SGI) is vital for predicting anthropogenic effects on surface water bodies and underlying aquifers. However, large-scale water and nutrient flux studies rely on surface water or groundwater-focused models. This study aims to model the effect of urbanization, which is usually accompanied by high groundwater abstraction and surface water pollution, particularly in the developing world, on a regional-scale SGI and nitrate loading. In the study area, the urban expansion increased by over 3% in the last decade. The integrated SWAT-MODFLOW model, Soil and Water Assessment Tool (SWAT) and Modular Finite-Difference Groundwater Flow (MODFLOW) coupling code, was used to assess SGI. By coupling SWAT-MODFLOW with Reactive Transport in 3-Dimensions, the nutrient loading to the river from point and non-point sources was also modeled. Basin average annual results show that groundwater discharge declined with increasing groundwater abstraction and increased with Land use/Land cover (LULC) changes. Groundwater recharge decreased significantly in the Belge season (February to May), and the river seepage and groundwater discharge decreased correspondingly. High spatiotemporal changes in SGI and nitrate loading were found under the combined LULC and groundwater abstraction scenarios. The water yield decreased by 15%. In a large part of the region, the nitrate loading increased by 17–250%. Seasonally controlled groundwater abstraction and water quality monitoring are essential in this region.

## Introduction

Water supply demand and water resources pollution are consistently increasing related to population growth, economic development, and lifestyle changes^1^. Urbanization and extensive agricultural activities emerge as leading water resources affecting anthropogenic factors^2–5^. Consequently, eutrophication became the ubiquitous problem of this era^5–7^. Usually, land cover changes increase the source of nitrate while decreasing the sink, and the nitrate exports are elevated in suburban and urban areas compared to the natural environment^2,8^. Nutrient sources and the exchange across the complex surface ad subsurface aquatic boundaries of urban and agricultural areas need to be understood to establish pollution control or correcting approaches^8,9^. Furthermore, the impacts of urbanization on groundwater systems must be explored and considered in land use planning to make future urban areas sustainable^3^.

Nitrate sources in the urban area include leaky sanitary sewers, industrial spills, leaching from landfills, stormwater runoff from roads, fertilizer use on lawns, sewage disposal, septic tanks, and infiltration from the polluted river^8,10,11^. The impact of the majority of these pollution sources on water resources is addressed^11^ but the latter, infiltration from polluted river networks, is ignored. Urban runoff is a widely known non-point type of pollution source in urbanized river basins^8,11,12^. Septic tank is a frequently cited high concentration nitrate source in urban groundwater, especially in developing countries, often with high unsewered slums^11^.

Addressing non-point sources of nutrients comprehensively in agricultural areas is an ongoing concern^8^. And several integrated regional-scale surfaces and subsurface models were developed and applied for evaluating nutrient loading in agricultural river basins, e.g.,^13–16^. However, concentration differences in aquifers underlying agricultural and urban areas are negligible and sometimes higher in urban regions because of the high number, density, and concentration of potential sources^11^. Thus, to prevent the adverse effects of urbanization on water resources, it is essential to assess the changes in groundwater flow and contaminant transport related to pollution in urban aquifers^17^. In river basins with hydraulically connected aquifer and surface water systems, the nutrient loading is complex and needs insight into surface water-groundwater interactions (SGI) to characterize the pollution^18–20^. Understanding regional-scale SGI, including the controlling factors, is also vital for conjunctive use of surface water and groundwater resources, assessment, and control of water contamination, and sustenance of wetlands^21–23^. However, human activities, especially groundwater over-abstraction and spatiotemporal recharge change associated with Land use/Land cover (LULC) changes, continuously alter the SGI distribution^3,24^. An SGI study is data extensive and often requires a multidisciplinary approach which makes it challenging for many data-scarce regions^9,19,25,26^.

These interlinked challenges of surface water and groundwater management in the changing environments require concerted efforts of hydrological, hydrogeological, and environmental scientists. Process-based coupled surface water-groundwater models appear to be plausible for simulating complex physical processes, such as urbanized and highly irrigated regions^12,26,27^. Moreover, integrated models provide a more accurate modeling approach, compared to separate saturated and unsaturated zones focused models, for best water resources management decisions^28^. Studies on nitrate loading into urban rivers by considering river-aquifer connection usually focus on small reach or experimental catchment, e.g.,^29^. This study focused on the simulation of urban expansion and groundwater pumping on the SGI and nutrient loading using an integrated modeling approach. This work incorporated non-point and point pollution sources with urban expansion scenarios and evaluated using an integrated surface-subsurface modeling approach. Of the many potential urban pollutants, we focused on nitrate because it is a common contaminant that can cause adverse health effects in infants and animals besides eutrophication^8,11,13,16^ and data scarcity in the study region.

The study region is in Africa. In Sub-Saharan Africa (SSA), where over 40% of the population lives in an urban area, wastewaters are often discharged untreated and increasingly become a leading source of eutrophication-causing nutrients^6,30^. In SSA, while population and urbanization have been increasing rapidly since 1950, most countries could treat only less than 30% of the total wastewater produced^6,30^. While regional-scale groundwater resources have been recently quantified, hydrogeological evidence at high resolution in many African urban centers is often lacking^30,31^. The fate and nutrient transport mechanisms in soils and aquifers and the effect of contaminants carried out from urban surfaces on nearby lakes and rivers, draining the urban areas, remain unexplored^6^.

## Study area descriptions

The study area is in Awash River Basin, Ethiopia. Awash River flows from central highlands to the northeast through the rift floor and ends in Lake Abhe, and the basin covers over 10% of Ethiopia's area. A significant part of the basin lies in the Great East African Rift Valley. Compared to other river basins in the country, Awash River Basin is the most developed basin^32,33^. The capital city and several small urban centers, urban-centered industries, small towns, and agricultural activities characterize the upper part of the basin. More than 60% of the country’s industries are established in the Awash River Basin^31,34^. Addis Ababa, Mojo, and Adama are among the major industrial areas in the region.

Since most of the industries were established following the river course, the fundamental water quality and quantity-related problems emanate from this arrangement^35^. A few industries have treatment plants^34^. For example, several industries in and around Addis Ababa discharge wastewater with limited or without treatment into the Akaki River^36^. Nevertheless, regardless of the anthropogenic pollution and over-abstraction in some parts of the basin, the water supply of the city depends entirely on the Akaki River Basin^37^. City expansion and population growth are escalating the demand; the contribution of groundwater to Addis Ababa’s water supply increased over three folds in the last 15 years^31,38,39^. Groundwater abstraction wells are scattered through the entire basin, including in the city, without protected areas^37^. Despite the vital role of the groundwater in the area, hydrogeological studies and data are extremely scarce or lack a systematic database that can easily be accessed^33,40^.

**Figure 1:**
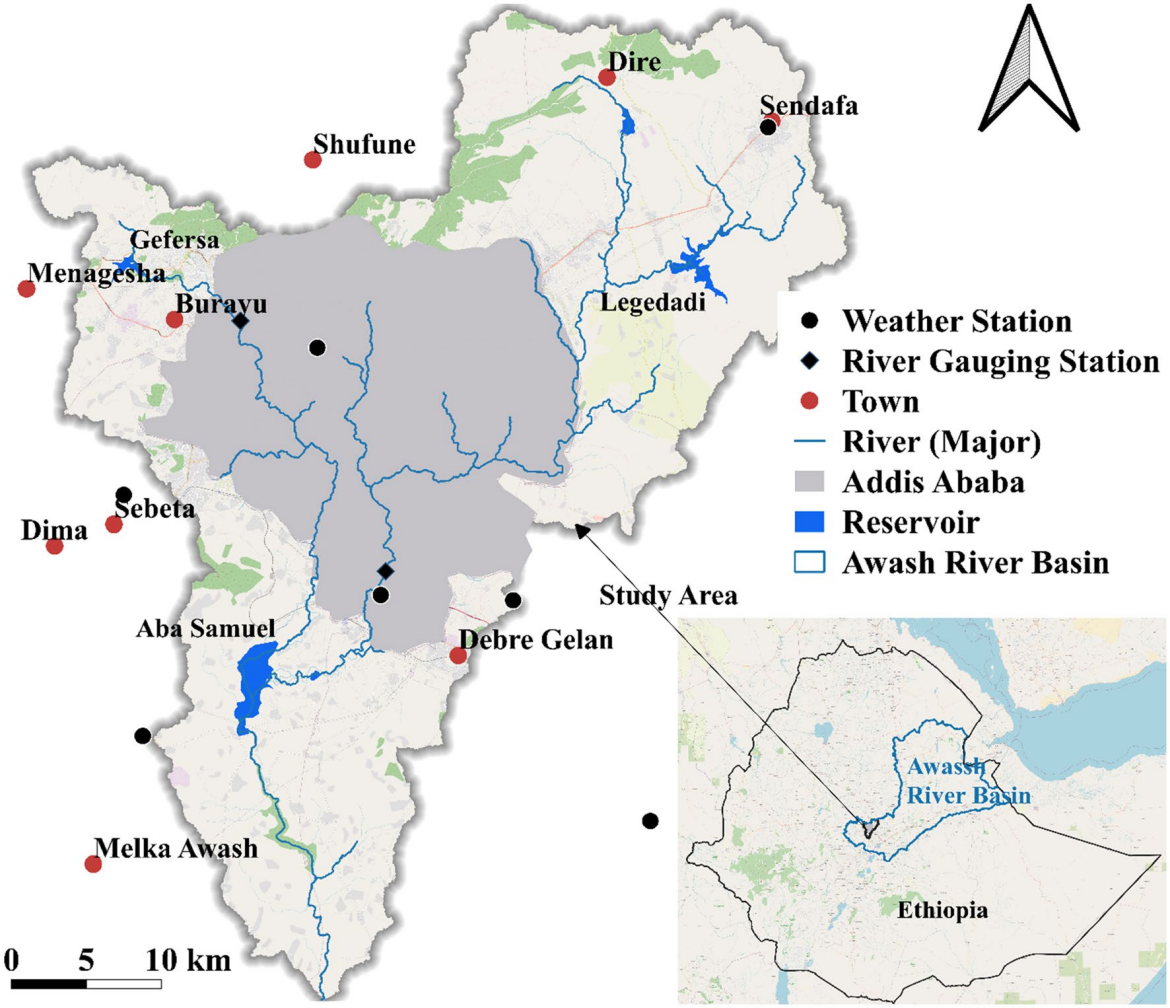
Description of the study area, including Major River networks, gauging stations, and reservoirs processed using QGIS3.10.4 (https://www.qgis.org/en/site/).

This study focuses on the most urbanized area of the Awash River Basin. The area is vastly developed, and consequently, the water resources’ quality and quantity, both surface and groundwater, are affected^35^. Reservoirs, namely Dire, Legedadi, Gefersa in the upstream area, and Aba Samuel downstream, are the main water bodies in the study area (Fig. 1). Gefersa and Dire reservoirs are relatively small. Aba Samuel reservoir served the city as a water supply source and hydropower generation. The rivers draining Addis Ababa transport high concentration pollutants, and the reservoir became a non-functioning swamp. The major rivers draining the region are known as Little Akaki, Kebena, and Big Akaki. Little Akaki River runs from Menagesha Mountain (upstream of Gefersa reservoir) to Aba Samuel. All three rivers drain the city, but Little Akaki crosses the most populated and industrialized part of the capital city.

Previously, the water supply of Addis Ababa derived from springs around the foot of the Entoto Ridge (northern part of the city) and dug wells in the central and southern parts of the city^38,41^. When the demand continued to increase, dams were constructed to use the rivers as a water supply source. Furthermore, exponential population growth and rapid urban expansion have led to further groundwater exploration in the Akaki well-field in the southern part of the city and several more places around the city. The continuous over-abstraction and lack of protection zone made the aquifer around Akaki well-field delicate^38^.

Waste management in Addis Ababa is in its infancy. The city has a poor sewer system and several residential and commercial buildings discharge their waste directly to the nearby river or runoff drainage network. The only municipal wastewater treatment plant is Kaliti Treatment Plant (KTP). The effluent from KTP is about 7500 m^3^/d with a peak value of 10,000 m^3^/d during rainy periods; it is a negligible amount for a city hosting over four million people ^6,38^.

The principal rainy season (Kiremt) in the region lasts from mid-June to September, while the dry season (Bega) is from October to January. In the rest of the months (Belge season), the area gets moderate and intermittent precipitation. In Kiremt, the region receives an average rainfall of about 759 mm and 200 mm in the Belge season. A warm temperate climate characterizes the area with an average temperature of 15 °C and relative humidity varying from 40 to 73%.

## Methods and materials

In this study, the Soil and Water Assessment Tool ^SWAT:^^42^, the Modular Three-Dimensional Finite-Difference Groundwater Flow ^MODFLOW:^^43^, and integrated SWAT-MODFLOW^44^ (version 2: https://swat.tamu.edu/software/swat-modflow/) models were employed. The SWAT-MODFLOW integrates SWAT and Newton formulation of MODFLOW-2005 ^MODFLOW-NWT:^^45^ in a single package. The SWAT model is widely applied to assess various river basin phenomena, including basin-scale water balances, the effect of LULC and climate changes on hydrological processes, and the assessment of point and non-point source pollution, mostly in agricultural areas^46^. MODFLOW-NWT is developed for solving drying and rewetting nonlinearities of groundwater flow in an unconfined system^45^. SWAT-MODFLOW was also integrated with the Reactive Transport in 3-Dimensions (RT3D) model to assess the nutrient loading^13^. This section highlights the models and setup, boundary conditions, and input data. QGIS3.10.4 (https://www.qgis.org/en/site/) and Python are used to prepare spatial maps and plot graphs.

### SWAT model inputs and setup

**Figure 2:**
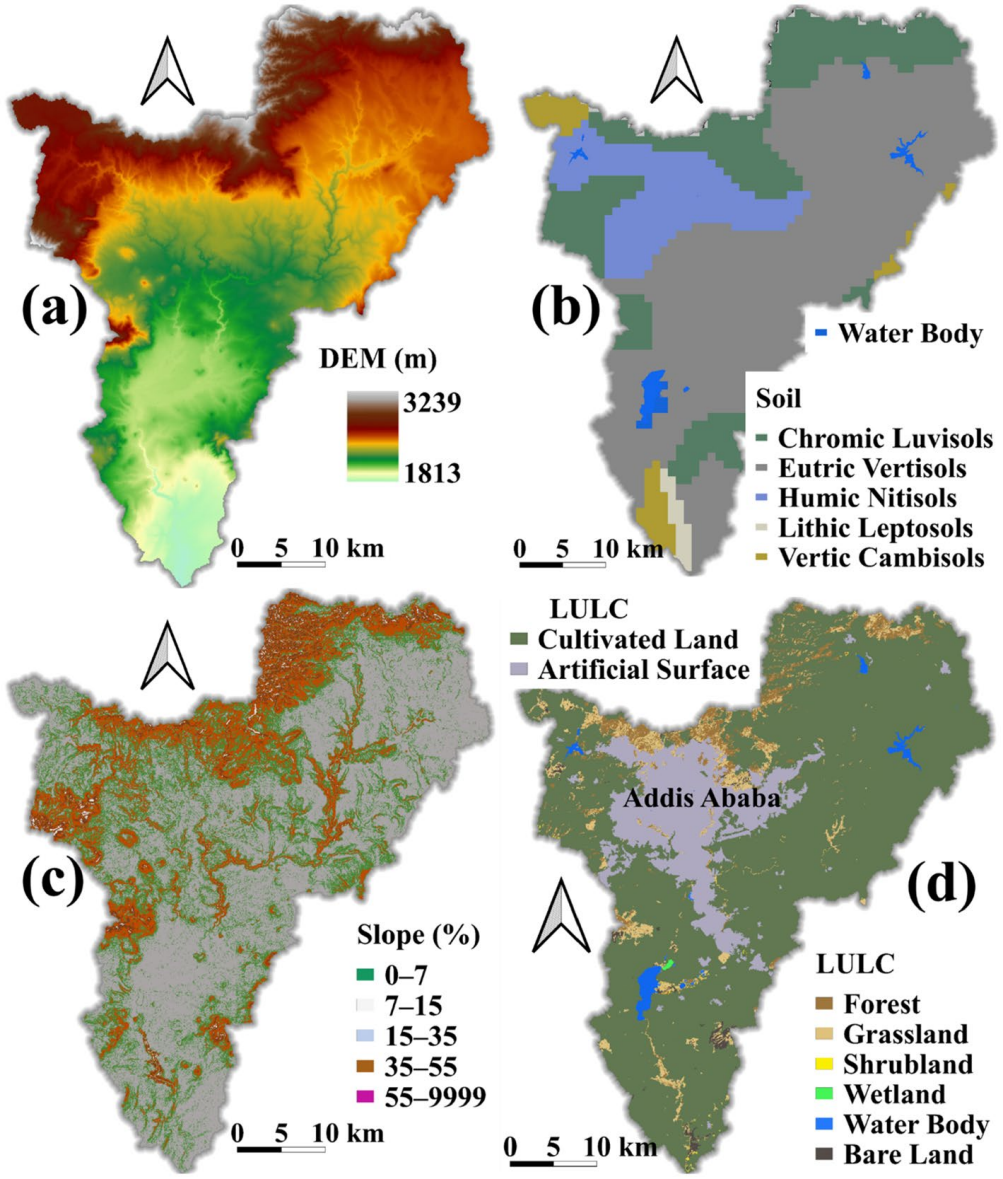
SWAT model inputs mapped using QGIS3.10.4 (https://www.qgis.org/en/site/): (**a**) Digital Elevation Model (DEM 30 m, USGS; https://earthexplorer.usgs.gov/), (**b**) Soil Classes^48^, (**c**) Slope classes, and (**d**) Land Use/Land cover (LULC) for the year 2000^47^.

The SWAT model setup needs LULC, soil properties, Digital Elevation Model (DEM), and daily climate data to simulate the fundamental hydrological processes. SWAT divides the model area (river basin) into subbasins and hydrologic response units (HRU). HRU represents the combination of unique LULC, soil properties, and slope classes. In this study, 30 m resolution LULC (for the years 2000, 2010, and 2020) and DEM accessed from GlobeLand30 provided by the National Geomatics Center of China^47^ and U.S. Geological Survey “earthexplorer” websites, respectively, were used (Fig. 2a, d). The GlobeLand30 LULC classification has ten types. However, in the study region only eight LULC classes, namely, Forest, Cultivated land, Grassland, Shrubland, Wetland, Water Bodies, Bareland, and Artificial surfaces (Table 1). The soil data were acquired from Harmonized World Soil Database v1.2^48^. Eutric Vertisols cover a large part of the area (Fig. 2b). Daily wind speed, temperature, relative humidity, precipitation, and solar radiation from 1980 to 2013 were collected from the National Metrological Agency of Ethiopia.

**Table 1:** Land use/Land cover classification and the percentage coverage from the total area for 2000, 2010, and 2020 in the study region.

LULC class	Description^47^	Decadal LULC (%)		
		2000	2010	2020
Cultivated land	Land used for cultivating crops	75.31	75.28	72.52
Forest	Forest area with a top density of over 30%	3.87	4.44	4.41
Grassland	Natural grass density over 10%	4.41	2.82	2.73
Shrubland	Shrubs with cover density over 30%	0.07	0.51	0.49
Wetland	Marshes, river floodplain, forest/shrub wetland, etc	0.41	0.54	0.54
Water bodies	Lake, river, reservoir, and pit-pond	0.58	0.43	0.33
Artificial surface	Mainly urban areas, transportation facilities	14.52	15.14	18.16
Bare land	Natural land with cover less than 10%	0.84	0.84	0.81

The study area, Akaki River Basin, was subdivided into 37 subbasins and an HRU of about 1363. The topography varies from 3239 m around Entoto Mountain to 1813 m above sea level near the outlet of the basin. About 77% of the area has a slope of 0–15% (Fig. 2c). The HRU was generated using the zero-threshold value of LULC, soil, and slope option.

### Groundwater flow model and hydrogeological descriptions

Topographically the study area favors the emergence of springs in the hills and valleys. The elevation descends from north to south, and more of the central, eastern, and southeastern parts are low areas covered with thick Quaternary deposits. Details of geology and hydrogeology data are important for building a representative model. However, like any other part of the country, data scarcity is the principal limiting factor for groundwater-related studies in particular. And therefore, the model developed for this study relied on prior studies^41,49,50^ and low resolution or short-duration data collected from the Ministry of Water Resources, Irrigation, and Electricity (EMWRIE) and Addis Ababa Water and Sewerage Authority (AAWSA).

Miocene–Pleistocene volcanic successions dominate the geology in Akaki River Basin, including acidic and intermediate lava flows, basaltic lava flows, and pyroclastic flows forming an interlayered sequence with quaternary faults^39^. The region has highly variable and complex aquifers. The aquifers in southern Addis Ababa are mainly young volcanic rocks of lava flows and tectonic fractures. The thickness and hydraulic conductivity of the unconsolidated sediments govern the subsurface infiltration, overlying weathered and fractured porous volcanic rocks with relatively high infiltration capacity, and favoring circulation and storage of subsurface water. Alluvial, residual, and lacustrine clay deposits dominate and vary with topography and geomorphology. The alluvial deposits, composed of clay with increasing thickness from north to south, are the predominant geological types along the rivers. A thin layer of residual clay soil covers the ridges and hillsides in and around the city. Thick residual clay soils cover high plains around the basin boundary. Black cotton lacustrine deposits cover the lower part of the region. In the area around the Akaki well-field, thick alluvial, lacustrine clay deposits overlay the underlying volcanic rocks interbedded between the volcanic rocks such as scoria, scoriaceous basalt, and basalts. As conceptualized by Ayenew et al.^49^ , the regional groundwater flow is from north to south. More details of the geology and hydrogeology of the basin are presented by several researchers, e.g.,^41,49^.

**Figure 3:**
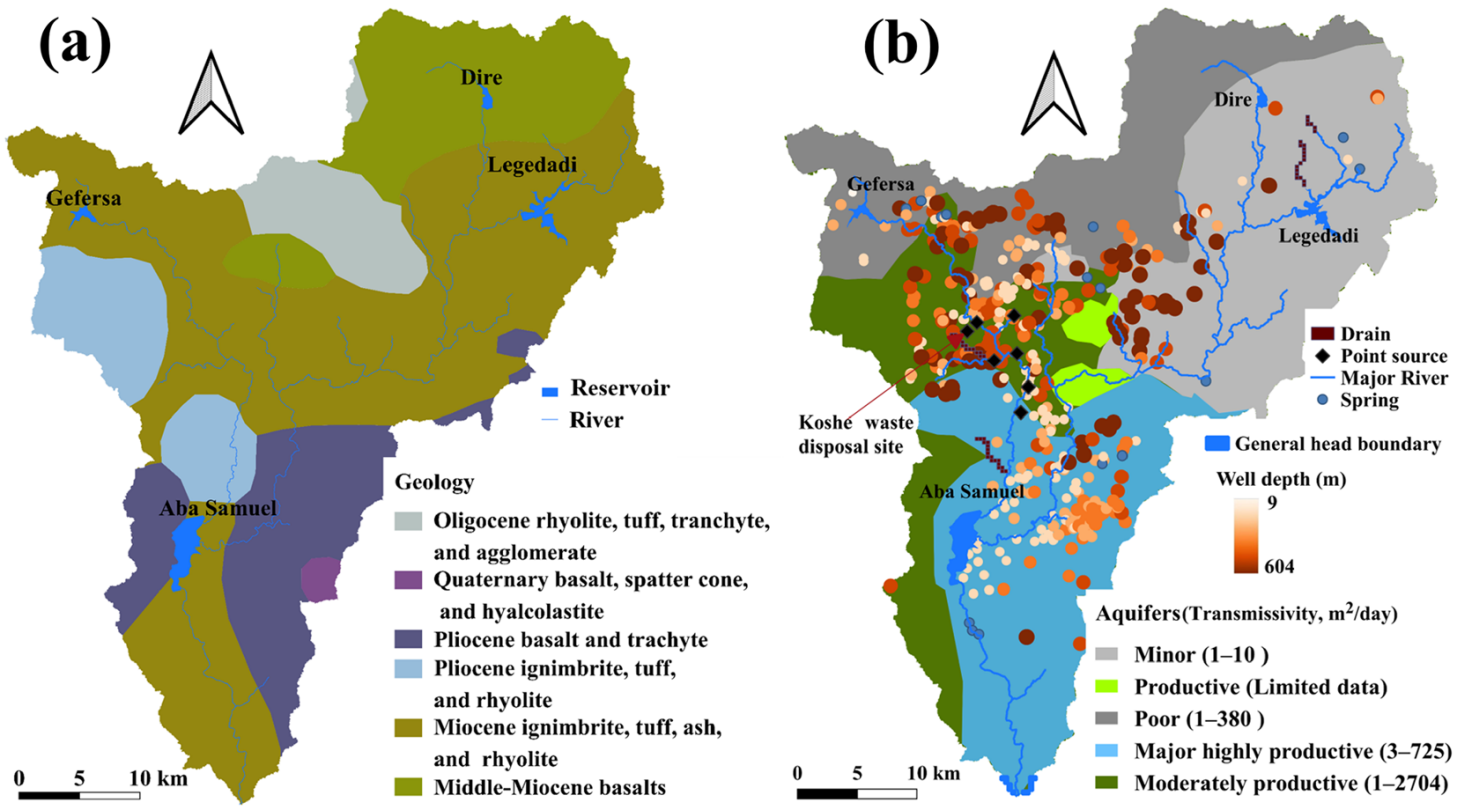
Geology and hydrogeology of Akaki River Basin, obtained from the Ministry of Water Resources, Irrigation, and Electricity (EMWRIE), the principal boundary conditions considered in the groundwater flow modeling, and location of contaminant point sources, processed using QGIS3.10.4 (https://www.qgis.org/en/site/).

The central volcanic hills and mountains, not crossed by faults, bound the river basin; the hydraulic conductivity is highly variable, 0.01–550 m/day^49^. The primary geologic classes in the basin are basalts, scoria, rhyolites, trachytes, ignimbrites, trachybasalts, and tuff of varying ages (Fig. 3a). The scoria and scoriaceous basalts with primary porosity and permeability make up highly productive aquifers in the region. The highly weathered and fractured basalts, ignimbrite, fractured tuff, and pyroclastics are also highly productive aquifers with secondary porosity and permeability. Fractured basalts, sparsely spaced joints and vesicles, ignimbrites, and agglomerates form moderately productive aquifers. The transmissivity is also highly variable, increasing from the highlands to Akaki well-field^41,49^.

Even though the study area is data-scarce, the hydrogeology framework is described in several previous works, e.g.,^49,51^. Previously, the groundwater flow modeling was performed by using two vertical layers and 400 × 400 m cell dimensions^49^**.** However, the thickness of the top layer is thin compared to the bottom layer, it varies from “a few meters to 53 m”^49^. In this study, it was found that the data which identify the thickness of the first layer was insufficient to build a reasonable model. And thus, a simplified convertible single-layer model was built. The mode area was discretized into 300 × 300 m horizontal resolution, which results in 18,976 active cells. The top and bottom boundaries were interpolated from the well-completion reports. The most important groundwater recharge-discharge areas (boundary conditions) are groundwater pumping, springs, reservoirs, river and canal networks, and percolation from precipitation. Even though pumping wells are distributed all over the basin, the primary groundwater pumping area is the Akaki well-field. In the area, pumping wells with varying depths up to 604 m below the surface and a pumping rate of 13,193 m^3^/day were identified and incorporated into the model using the Well Package. Details of the reservoir operation are unavailable, and thus, to simplify the data requirements, it was modeled as General Head Boundary. The major rivers were imported from the SWAT model and modeled using the River Package. Canal or small rivers in a few places were modeled using Drain Package (Fig. 3b). The initial groundwater head was interpolated from the static water level record.

### Integrated SWAT-MODFLOW model

Both SWAT and MODFLOW models are widely used and well known among hydrologists and hydrogeologists. And therefore, here, the general equation of SWAT and MODFLOW models and a brief description of the integrated SWAT-MODFLOW code are given (Eqs. 1 and 2). The general equation of the SWAT model is as follows^42^:

1 $$SW_{t} = SW_{0} + \mathop \sum \limits_{i = 1}^{t} (PCP - Q_{s} - ET - Perc - Q_{gw} )$$

where *SW*_*o*_ and *SW*_*t*_ are the quantities of initial and final soil water (mm/day), respectively, *t* is the time (days), *PCP* is the precipitation (mm/day), *Q*_*s*_ is the surface runoff (mm/day), *ET* is the evapotranspiration (mm/day), *Perc* is the percolation (mm/day), and *Q*_*gw*_ is the flow from the aquifer (mm/day).

MODFLOW is physically-based algorithm that solves groundwater flow through porous earth material^43,52^. The model is based on the following general partial differential equation ^43^:

2 $$\frac{\partial }{\partial x}\left( {K_{xx} \frac{\partial h}{{\partial x}}} \right) + \frac{\partial }{\partial y}\left( {K_{yy} \frac{\partial h}{{\partial y}}} \right) + \frac{\partial }{\partial z}\left( {K_{zz} \frac{\partial h}{{\partial z}}} \right) \pm W = S_{s} \frac{\partial h}{{\partial t}}$$

where *K*_*xx*_, *K*_*yy*_, and *K*_*zz*_ are principal components of the hydraulic conductivity tensor in the *x*, *y*, and *z* special directions, *W* is the source or sink, *S*_*s*_ is the specific storage (1/L), *h* is the hydraulic head (L), and *t* is time.

Among the advantages of using the integrated SWAT-MODFLOW is water yield computation. The basic water yield calculation in the SWAT model is based on the following equation ^42^:

3 $${\text{WY}} = {\text{Q}}_{{\text{s}}} + {\text{Q}}_{{{\text{gw}}}} + {\text{Q}}_{{{\text{lat}}}} - {\text{T}}_{{{\text{loss}}}}$$

where WY is water yield (L^3^/T), Q_s_ is surface runoff (L^3^/T), Q_gw_ is groundwater flow to the river (L^3^/T), Q_lat_ is lateral soil flow (L^3^/T), T_loss_ is water loss through the riverbed (L^3^/T). T_loss_, in Eq. 3, is computed with an assumption that when a channel does not receive groundwater, possibly it loses water through the side and bed and joins bank storage or the deep aquifer and approximated using the following relationships:

4 $${\text{T}}_{{{\text{loss}}}} = {\text{K}}_{{{\text{ch}}}} .{\text{T}}_{{\text{Q}}} .{\text{P}}_{{{\text{ch}}}} .{\text{L}}_{{{\text{ch}}}}$$

where K_ch_ is the effective hydraulic conductivity of the channel alluvium (L/T), T_Q_ is the flow travel time (T), P_ch_ is wetted perimeter (L), and L_ch_ is channel length (L).

SWAT-MODFLOW uses the MODFLOW River Package that is formulated based on Darcy’s law to compute water loss from the river and groundwater discharge to the river with the following mathematical principle:

5 $${\text{Q}}_{leak} = {\text{K}}_{{{\text{bed}}}} \left( {{\text{L}}_{{{\text{ch}}}} .{\text{P}}_{{{\text{ch}}}} } \right)\left( {\frac{{{\text{h}}_{{{\text{ch}}}} - {\text{h}}_{{{\text{gw}}}} }}{{\text{M}}}} \right)$$

where ${\mathrm{Q}}_{leak}$ is volumetric water flux between the river and the aquifer (L^3^/T); K_bed_ is hydraulic conductivity of the riverbed (L/T); M is riverbed thickness (L); h_ch_ and h_gw_ are river stage and groundwater head (L), respectively; P_ch_ is wetted perimeter (L); L_ch_ is channel length (L). Equation 5 shows that if the groundwater head is higher than the water level in the channel, ${\mathrm{Q}}_{leak}$ will be negative, which means water flows from the aquifer to the channel, and ${\mathrm{Q}}_{leak}$ will be positive if the reverse is true. In SWAT-MODFLOW water yield computation is based on ${\mathrm{Q}}_{leak}$ (Eq. 5) instead of T_loss_ (Eq. 4).

The recent SWAT-MODFLOW algorithm^13,27,44^ was used to integrate the calibrated SWAT-2012 and Newton formulated MODFLOW-2005 ^MODFLOW-NWT:^^45^. The current version of SWAT-MODFLOW^44^ has been applied to study several hydrological processes, including groundwater recharge^26^, regional-scale SGI^44,53,54^, and the effect of natural and anthropogenic factors on water balance^55–60^. However, the diversity of application regions and study objectives is limited compared to the individual SWAT and MODFLOW models.

### Reactive transport in 3-dimensions (RT3D) model

Recently, Wei et al.^13^ applied SWAT-MODFLOW for the assessment of non-point source contaminant transport in agricultural areas by integrating it with Reactive Transport in 3-Dimensions ^RT3D;^^61^. RT3D is a contaminant and solute transport model that can simulate dispersion, advection, and chemical reactions in a saturated groundwater system^61^. The algorithm has the functionality of solving multi-species reactive transport. The following governing equation describes the fate and transport of aqueous and solid-phase species in multi-dimension saturate porous media^61^:

6 $$\phi \frac{{\partial C_{k} }}{\partial t} + \rho_{b} \frac{{\partial \hat{C}_{k} }}{\partial t} = \frac{\partial }{{\partial x_{i} }}\left( {\phi D_{ij} \frac{{\partial C_{k} }}{{\partial x_{j} }}} \right) - \frac{\partial }{\partial xi}\left( {\phi v_{i} C_{k} } \right) + q_{s} C_{{s_{k} }} + \phi r_{c} , {\text{where k}} = {1},{ 2}, \, \ldots ,{\text{ m}}$$

In Eq. 6, m is the total number of species, ${C}_{k}$ is the concentration of the k^th^ species (M/L^3^), t is time (T), ${D}_{ij}$ in the dynamic dispersion coefficient tensor (L^2^/T), x_i_ is the distance along the respective axis coordinate (L), v_i_ average seepage velocity (L/T), ϕ is the porosity of the material, ${q}_{s}$ is the volumetric flux of water per unit volume of aquifer representing sources and sinks (1/T), ${C}_{{s}_{k}}$ is the concentration of the k^th^ species in the sources or sinks (M/L^3^), ${\rho }_{b}$ is bulk density of porous media (M/L^3^), ${\widehat{C}}_{k}$ is the solid-phase concentration of the k^th^ species (M/M), ${r}_{c}$ the rate of all reactions that occur in the aqueous phase (ML^3^/T).

RT3D uses the groundwater hydraulic head, cell-by-cell flow data, and water sources and sinks simulated by the MODFLOW model to establish the groundwater flow field. The detailed linkage processes with SWAT-MODFLOW are provided in ^13,16,61^. SWAT-MODFLOW-RT3D combines SWAT-MODFLOW code as a base and RT3D as a sub-routine into a single executable algorithm^13^. The integrated model uses the SWAT ‘in-stream’ algorithm to rout nitrate through the stream network. In SWAT-MODFLOW, the SWAT model computed recharge is mapped to MODFLOW grid cells, and MODFLOW simulates the groundwater flux in each cell^61^. RT3D uses MODFLOW simulated groundwater flux. RT3D also receives the nitrate concentration from the SWAT model recharge water and river network and computes the change in each grid cell and river mass loading.

**Figure 4:**
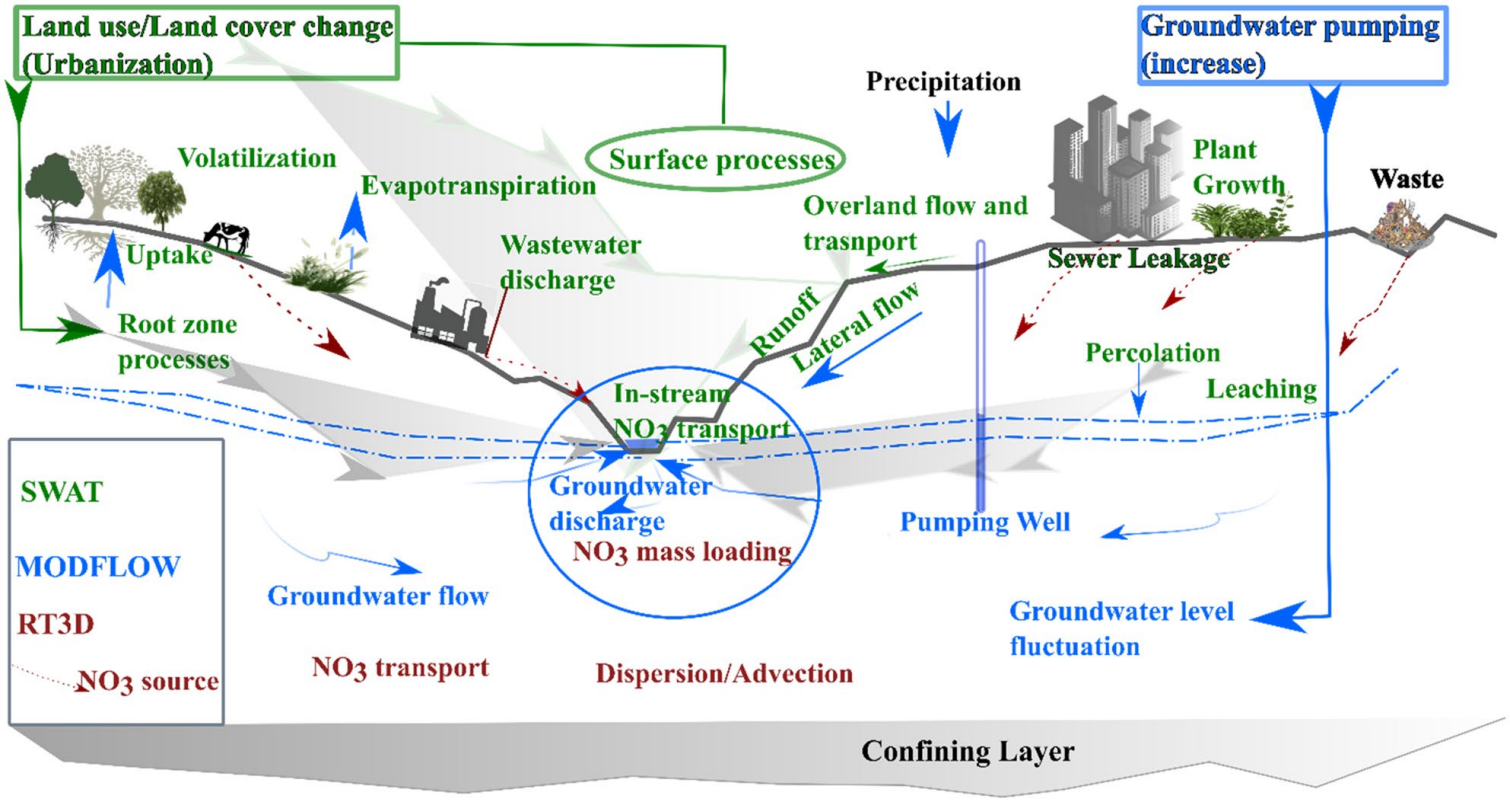
Conceptual diagram showing surface and subsurface processes and modeling framework, adopted and modified from previous studies^13,16,44^, with copyright permission.

In the study area, the major point sources of nitrates are industrial discharges into the river and leaching from waste disposal sites ^39,62^. Sources with high concentrations such as industrial discharge and leaching from solid waste disposal sites ^63^, along Little Akaki River, were selected (Fig. 3b). In these sites as identified by several researchers, e.g., Angello et al. ^63^, the nitrate concentration varies from 33 mg/l to 300 gm/L. The biggest waste disposal site called Koshe was modeled as constant concertation (300 mg/L) point source. The denitrification is specified using single-Monod expression so that the reaction rate depends on the nitrate presence ^64^. Thus, the values of the first order-rate denitrification constant (1/d) and Monod half-saturation constant (M/L^3^) are required for the model setup. However, these values are not easy to measure and are rarely available for watershed-scale studies, usually assumed based on commonly used values from the literature. In this study area, related studies are unavailable, and therefore, we have referred to worldwide literature, e.g., ^15,16,64–66^, and considered 0.05/day and 10 g/m^3^ for the first-order denitrification and Monod half-saturation constant. The surface and subsurface hydrological processes, models, and scenario simulations were conceptualized by modifying the schematic diagram from previous studies ^13,16,44^. Different colors are used to identify the processes and the corresponding simulating algorithm (Fig. 4).

### Model calibration and validation

The SWAT model was calibrated and validated using sequential uncertainty fitting version 2 (SUFI-2) in the SWAT-CUP^67,68^ user interface. Monthly river flow at two gauging stations, Little Akaki and Big Akaki, obtained from EMWRIE was used. The calibration and validation performance was evaluated using the Nash–Sutcliffe Efficiency coefficient (NSE) and minimizing the Percent Bias (PBIAS), Root Mean Square Error (RMSE), and coefficient of determination (R^2^).

The groundwater model was also calibrated using the Parameter ESTimation (PEST) Package. However, the automatic calibration technique was limited to the steady-state simulation because of the transient groundwater level data scarcity. The important calibrated parameters were hydraulic conductivity, river conductance, and drain conductance. The zonal and pilot point techniques^69^ were used to calibrate the hydraulic conductivity using a range value of 0.012 to 375 m/day. The initial groundwater head is a key input for the simulation of integrated surface water and groundwater flow models. In this study, the initial head was based on the calibrated steady-state MODFLOW model simulation. Nevertheless, transient simulation parameters (specific storage, specific yield) of the aquifer calibrated after integration using a trial-and-error technique based on the groundwater level fluctuation, groundwater discharge (or comparing the measured and simulated river flow), and the overall water balance of the study area. In addition to these soft calibration and validation practices previous studies in the region, e.g.,^41,49,70,71^, were important sources of initial ranges of parameters and were also used as a benchmark for general the model outputs analogy.

Automatic calibration functions are unavailable in the recent version of SWAT-MODFLOW. Individually calibrated SWAT and MODFLOW for Akaki River Basin were integrated, and moderate manual calibration was performed (Table 2). In SWAT-MODFLOW-RT3D mainly longitudinal dispersivity and initial concentration of nitrate in groundwater were adjusted by trial and error. We have used longitudinal dispersivity of 120–180 m and constant porosity of 0.3.

**Table 2:** Initial range, calibrated value, and definition of parameters used in the calibration and validation processes.

Parameter	Description	Initial	Calibrated
r__CN2.mgt	Soil Conservation Service (SCS) runoff curve number	− 0.2–0.5	0.1 and 0.15
v__GW_DELAY.gw	Groundwater delay (days)	0–500	2.5
v__RCHRG_DP.gw	Deep aquifer percolation fraction	0–1	0.33
v__ALPHA_BF.gw	Baseflow recession factor	0–1	0.06
v__REVAPMN.gw	Threshold water depth to initiate percolation (mm H_2_O)	0–500	105.53
v__ESCO.hru	Soil evaporation demand coefficient	0–1	0.85
r__SOL_AWC.sol	Soil available moisture capacity (mm H2O/mm soil)	− 0.5–0.5	0.25
	Specific yield (%)	2–30	11–27
	Specific storage (m^-1^)	–	1 × 10^–5^

r__default SWAT parameter is multiplied by (1 + optimized value); v__ replaced the default value.

### Simulation scenarios

Two important water and nutrient mass balance affecting factors, namely LULC changes focused on the urban expansion and groundwater pumping for urban water supply, were tested using an integrated modeling approach. The effects were evaluated based on the baseline simulation. Therefore, the simulations were baseline, the effect of groundwater pumping (scenario1), urban expansion in the period of 2000–2010 (scenario 2), urban expansion from 2000 to 2010 and groundwater pumping (scenario 3), urban expansion from 2010 to 2020 (scenario 4), and urban expansion between 2010 and 2020 and groundwater pumping (scenario 5). The LULC data is tabulated in “Groundwater flow model and hydrogeological descriptions” Sections (Table 1). The current groundwater pumping rate was used as a reference and increased by 15–35% to evaluate the effect (Table 3). Related scenarios were simulated in a previous study^49^ to assess the aquifer response using MODFLOW. Under all scenarios formulated and simulated in this study, the regional-scale SGI, groundwater recharge, and nutrient loading were the focus of the analysis.

**Table 3:** Detail of groundwater pumping wells based on hydrogeology map of the study area: Well depth, pumping rate, number of wells located in each hydrogeologic zone, and pumping rate change (%).

Aquifer	Well depth	Pumping (m^3^/day)	Number	Change (%)
Minor	94–400	138.24–2,937.6	58	15
Productive	–	–	–	–
Poor	9–250	21.6–4,060	88	15
Moderately productive	47–257	34.56–2,592	146	25
Major highly productive	53–604	43.2–13,193	151	35

## Results and discussion

In 2000, the urban coverage was 14.52% of the total study area. The LULC change assessment shows that the urbanization from 2000 to 2010 was trivial compared to the recent expansion. The Grassland and Artificial surfaces were the most affected classes. From 2010 to 2020, the Grassland decreased by around 1.7%, while the urban area extended by about 3%. The Forrest and Shrubland coverage showed a slight improvement. The effect of these changes on the spatiotemporal distribution of SGI and nitrate seepage from the river to the aquifer is presented compared to the baseline simulation.

### Baseline simulation results

The baseline simulation results were analyzed after the calibration and validation of the model. The statistical model evaluation using river flow shows an NSE value of 0.68–0.82, R^2^ 0.84–0.89, and PBIAS − 0.27 to − 9.14 (Table 4).

**Table 4:** SWAT-MODFLOW model calibration and validation performance indicating statistical values.

Subbasin (gauging station)	Calibration			Validation		
	R^2^	NSE	PBIAS (%)	R^2^	NSE	PBIAS (%)
Big Akaki	0.85	0.68	− 1.12	0.84	0.72	− 0.27
Little Akaki	0.89	0.82	− 0.73	0.86	0.72	− 9.14

**Figure 5:**
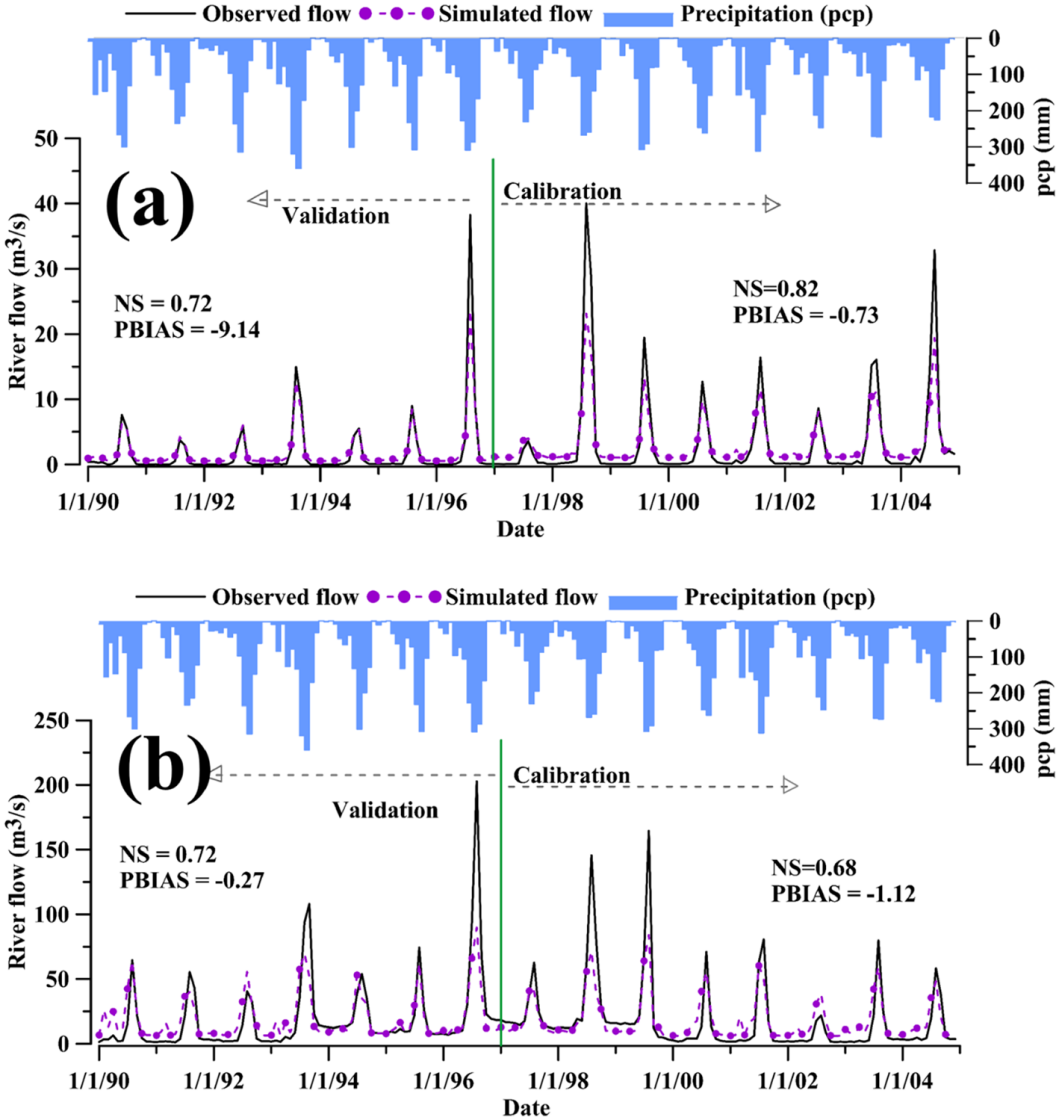
Calibration and validation of river flow at Little Akaki (**a**) and Big Akaki (**b**) gauging stations and the average precipitation in the study area.

The model performance based on the criteria proposed by Moriasi et al.^72^ ranges from “good” to “very good”. The peaks and lows of the flow match well with the corresponding basin average precipitation. However, the performance at low and peak flows was dissimilar in the calibration and validation period. The model overestimated the low flows and underestimated the peak flows. For Little Akaki, the validation performance was less than the calibration, while for the Big Akaki, the validation was better than the calibration (Fig. 5). The SWAT-MODFLOW based river flow showed a slight improvement at low flow compared to the SWAT model. In Little Akaki, the flow slightly increased from 1997 onward compared to the preceding years, and rapid urbanization could be one reason for this.

**Figure 6:**
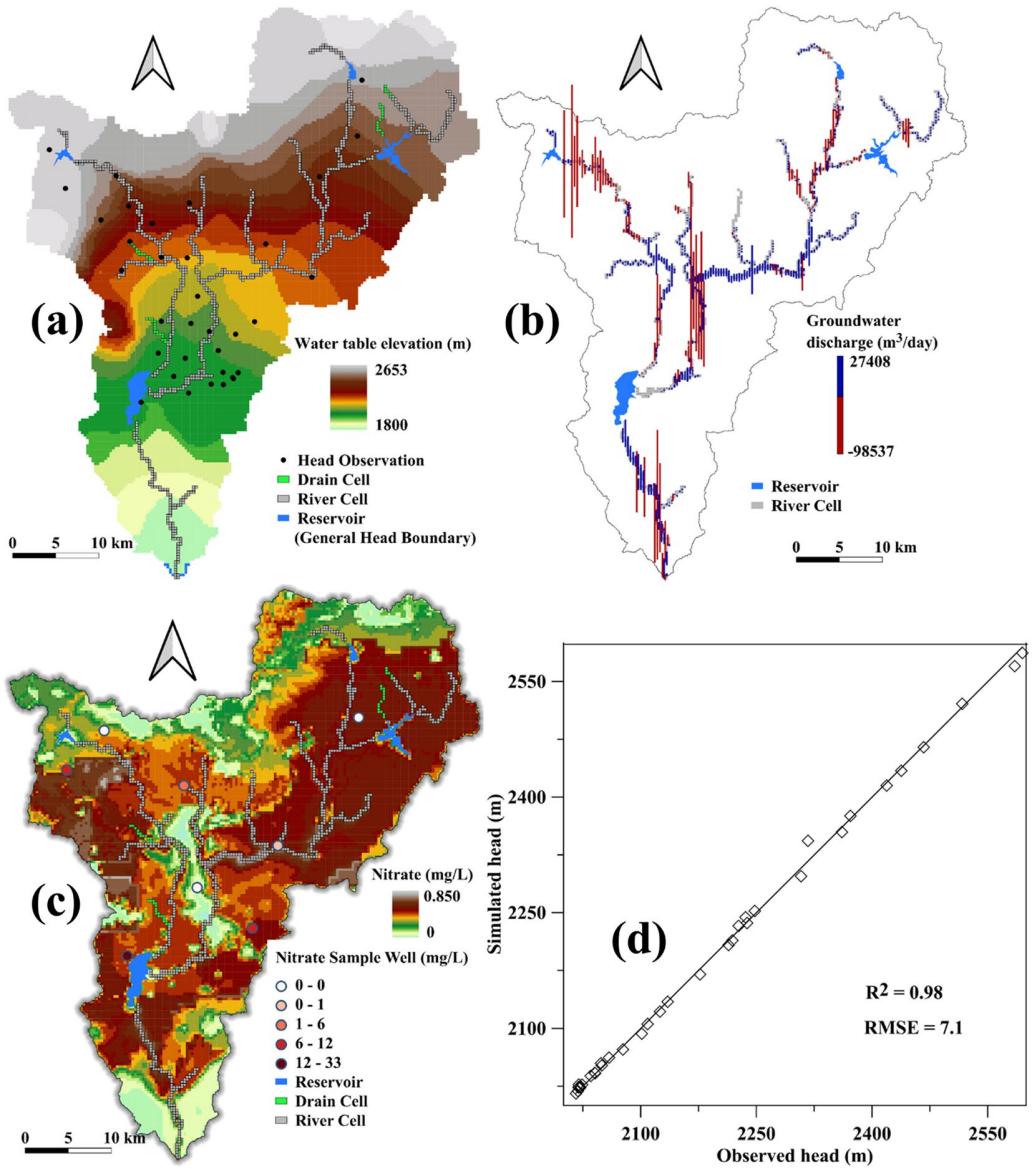
Map showing the calibrated starting groundwater head (**a**), the spatial distribution of groundwater discharge to the river (**b**), the initial nitrate concentration (**c**) in the Akaki Aquifers prepared using the QSWATMOD2 plugin (https://swat.tamu.edu/software/swat-modflow/), and scatter plot of measured and simulated groundwater head (**d**).

The calibrated groundwater head varied from 1800 to 2653 m (Fig. 6a). Previous research outputs^49^ present a closely related calibrated groundwater head. The simulated and observed head agreed fairly with an R^2^ value of 0.98 and RMSE of 7.1(Fig. 6d). The model underestimated the groundwater level in the altitude areas.

The average groundwater discharge to the river map (Fig. 6b) shows that in the study area, the groundwater discharge to the river occurred almost in all sections of the river. However, the seepage from the river to the aquifer was in limited areas, and the magnitude was significantly high, comparatively. The initial nitrate concentration in groundwater was also evaluated using a limited number of measurements obtained from the EMWRIE (Fig. 6c). The measured nitrate concentration varies from zero to 33 mg/L. The initial simulated concentration varied from zero to 0.9 mg/L. The spatial distribution of high and low measured and simulated concentration matches well with an RMSE value of 6.5, even though there is a difference at peak values. While, in a large part of the area, the measured and simulated data show good agreement, the main reason for the peak value of measured and simulated concentration mismatched is that the points with higher concentration have no spatial relation and are limited in number. The spatial distribution of simulated initial nitrate concentration represented the groundwater quality in the study area.

**Figure 7:**
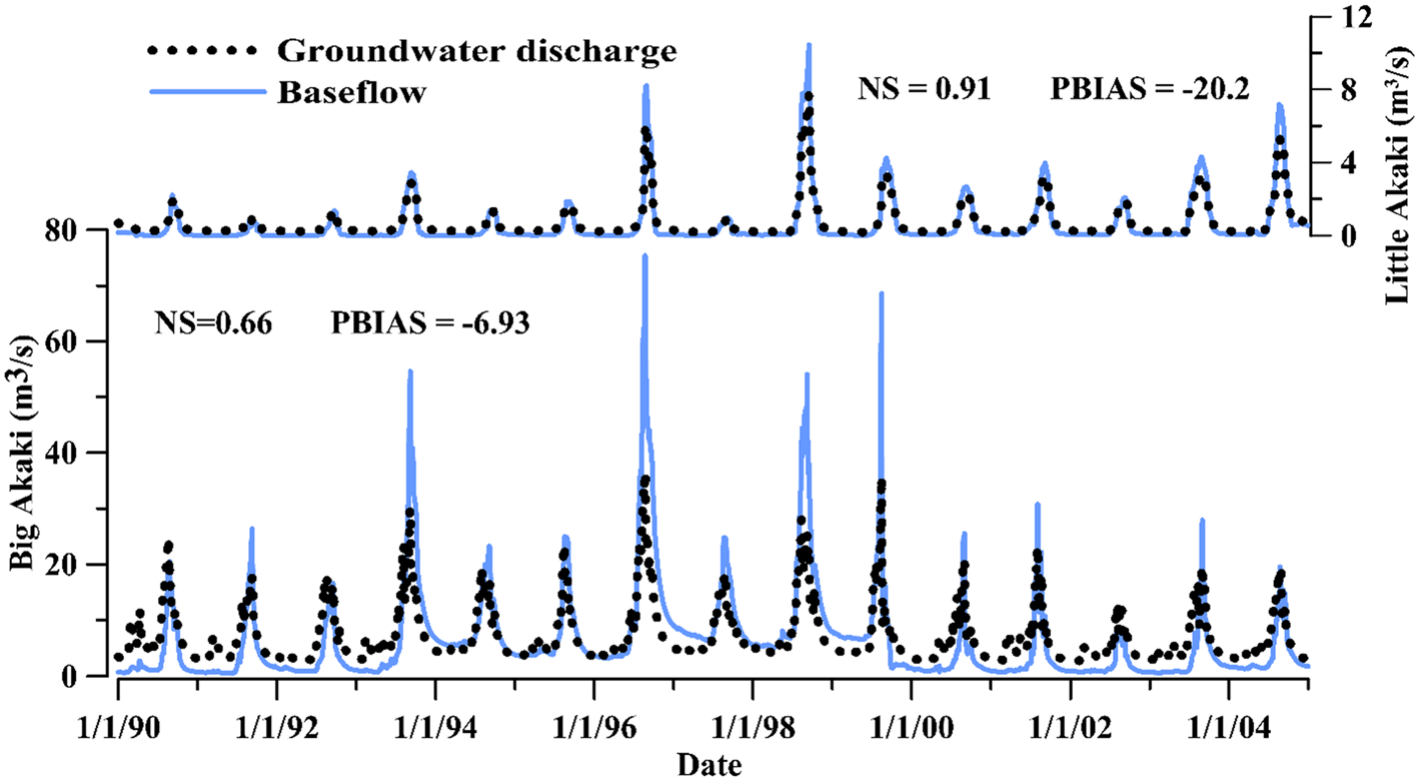
Comparison of SWAT-MODFLOW simulated groundwater discharge and baseflow in Little Akaki and Big Aaki Rivers.

To show the model performance in transient groundwater flow simulation (SWAT-MODFLOW), the groundwater discharge to the river and baseflow, analyzed from observed river flow using automated separation techniques^73^, were compared (Fig. 7). The baseflow and groundwater discharge matched fairly, but SWAT-MODFLOW underestimated the discharge in the rainy period and overestimated in dry periods.

The LULC change effect was substantial on average annual basin surface runoff (surq), recharge (rch), and lateral flow (latq). The change in lateral flow from the baseline was quite significant (~ 72%). The river seepage and groundwater discharge were controlled by both groundwater pumping and LULC changes (Table 5). Simulation results using the 2000 LULC data shows that river seepage increased under groundwater abstraction but decreased in all other scenarios. The seepage decreased by more than 4%. The groundwater discharge declined with the increasing groundwater pumping while increasing under the LULC change scenarios. This result is likely to be related to the moderate recharge increment under the LULC change scenarios.

**Table 5:** Principal annual basin water balance components, including precipitation (prec), surface runoff (surq), lateral flow (latq), groundwater discharge to the river (gwq), surface water discharge to the aquifer (swgw), and recharge (rch) of the study area under all simulation scenarios (mm).

Variable	Baseline	Scenario 1	Scenario 2	Scenario 3	Scenario 4	Scenario 5
prec	1006.24	1006.24	1006.24	1006.24	1006.24	1006.24
surq	344.70	344.70	297.76	297.76	294.79	294.79
latq	11.40	11.40	19.10	19.10	19.15	19.15
gwq	356.0	349.46	357.63	351.04	358.46	351.88
swgw	126.55	128.16	121.30	122.78	121.01	122.54
rch	166.14	166.14	169.83	169.83	170.31	170.31

### Surface water-groundwater interactions

The effect of the groundwater pumping and urban expansion on SGI in terms of groundwater recharge, river seepage, and water yield over the simulation period was immediately apparent. After several trial-and-error simulations, the effect of groundwater pumping, which increased by an average of the proposed increase rate from the baseline pumping (25%), was considered. The total groundwater abstraction in the baseline scenario was 99,000 m^3^/day.

**Figure 8:**
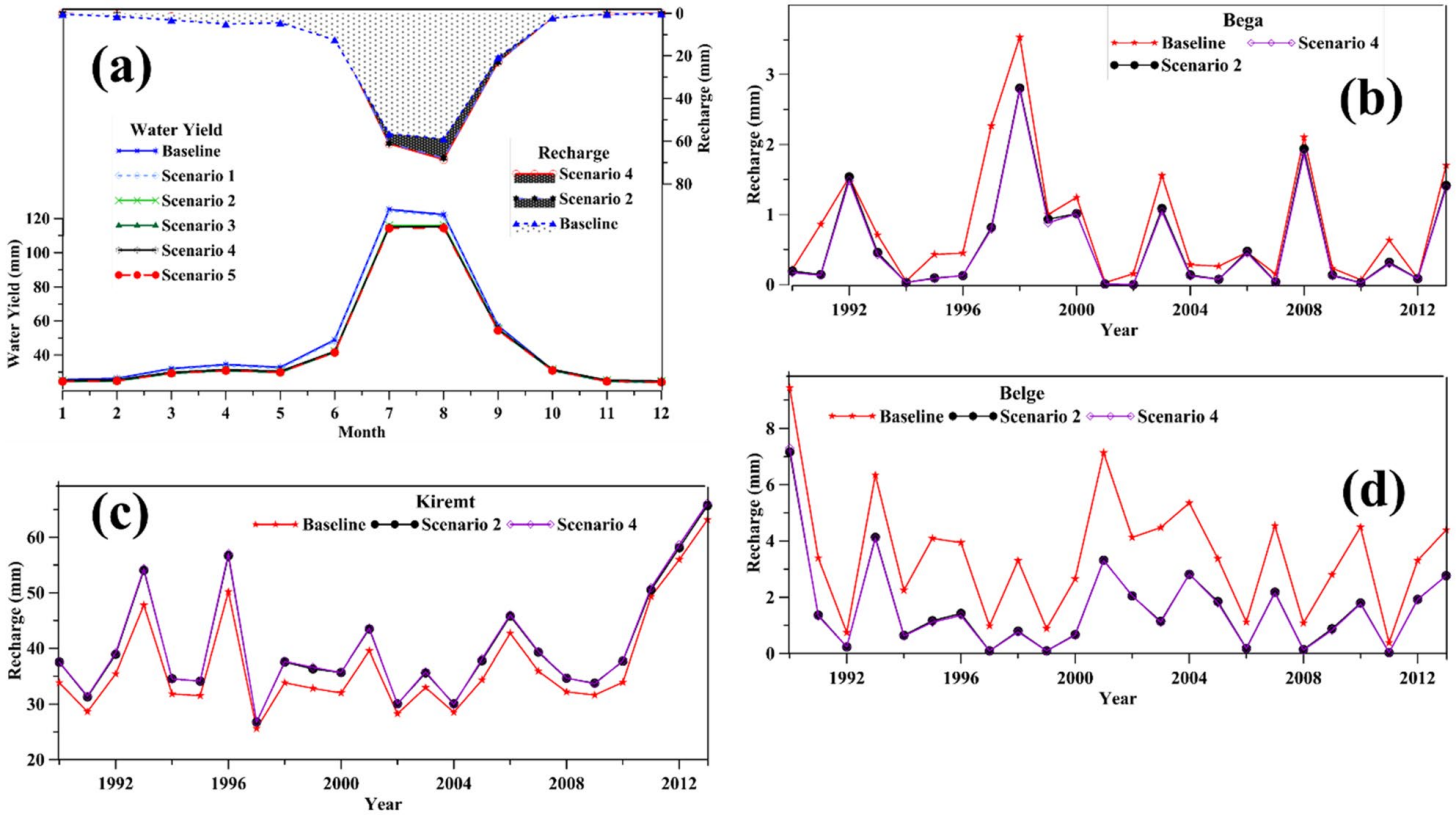
Average monthly groundwater recharge and water yield (**a**) and seasonal groundwater recharge fluctuation from 1990 to 2013 (**b**–**d**) in the Akaki River Basin.

The graphical comparison shows that the effect of groundwater pumping on the monthly water yield was insignificant (Fig. 8a). However, the average value revealed that groundwater pumping caused a decrease in water yield by up to 3%, the LULC change by 9%, and the combined LULC and groundwater pumping by up to 15%. The urban expansion caused to increase in the groundwater recharge in Kiremt and a decrease in the Bega and Belge seasons (Fig. 8b–d). Principally in the Belge season, the recharge showed a significant difference compared to the baseline simulation (Fig. 8d). The recharge under scenarios 2 and 4 had an insignificant difference in all seasons. These results further support the effect of rapid urbanization that occurred from 2010 to 2020 compared to the preceding decade.

**Figure 9:**
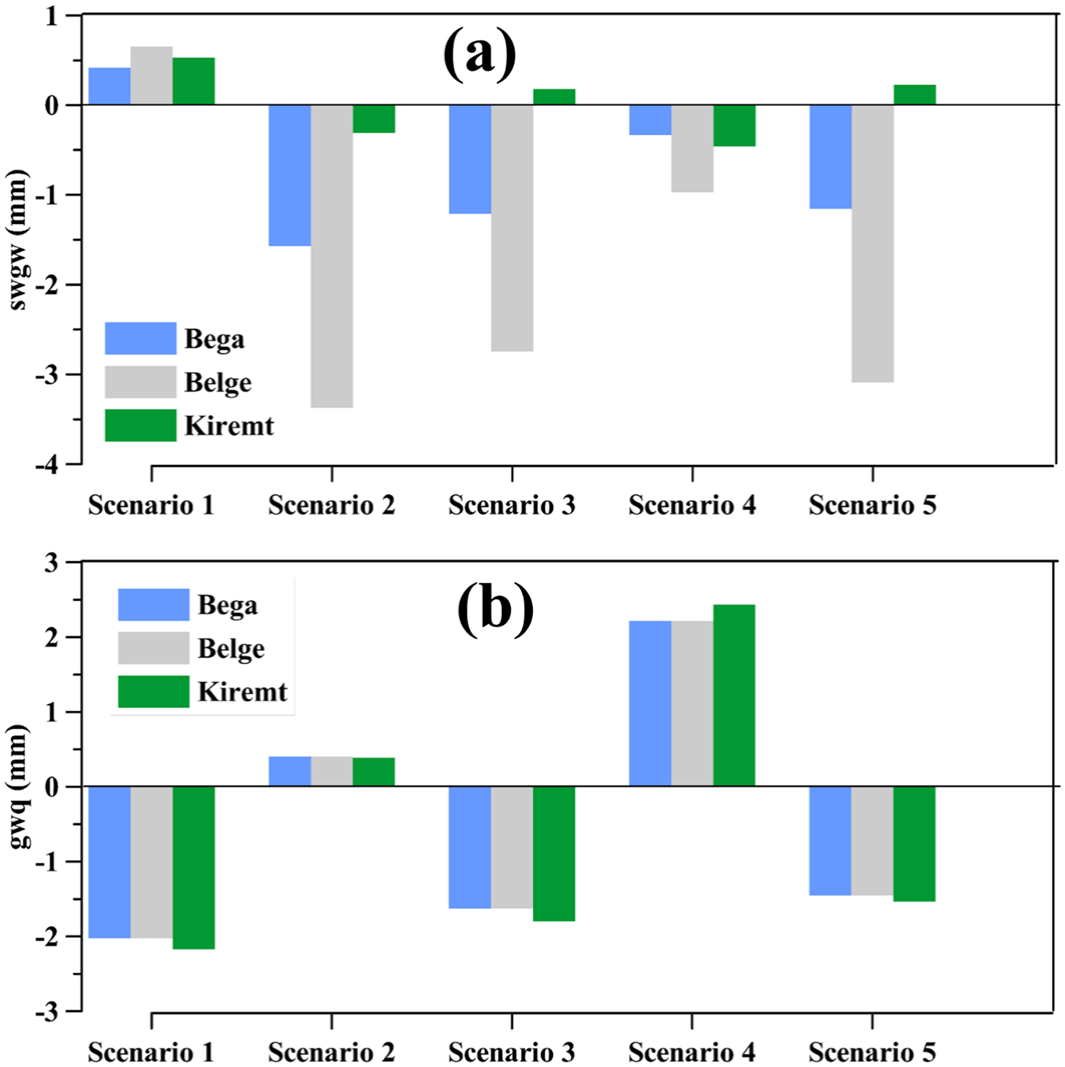
Seasonal surface water discharge to the aquifer (swgw) and groundwater discharge to the river (gwq) differences under the simulation scenarios from the baseline scenario (scenarios-bassline).

Maximum river seepage and groundwater discharge occurred in Belge and Kiremt, respectively (Fig. 9). The LULC change had a decreasing effect on river seepage (Fig. 9a); the effect was significant in the Belge season. On the other hand, the groundwater discharge increased with LULC change (Fig. 9b, scenarios 2 and 4). The groundwater discharge difference between the seasons was trivial.

**Figure 10:**
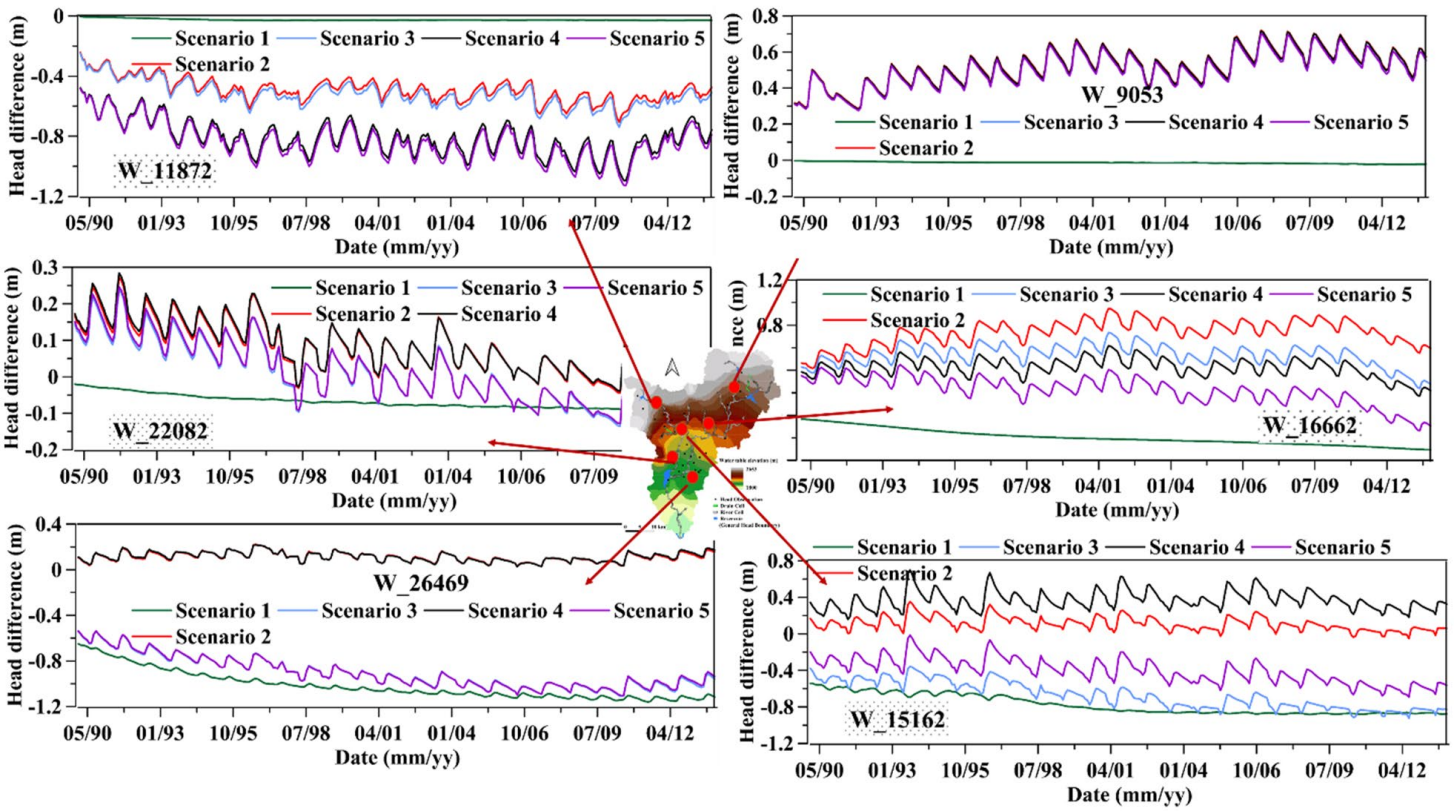
Groundwater level difference from the baseline scenario from 1990 to 2013: positive values show that the groundwater level decreased compared to the baseline simulation.

Figure 10 shows the groundwater head differences from the baseline simulation at selected wells. The fluctuation was significant around Akaki well-field. The spatiotemporal groundwater level variability under all scenarios (difference from the baseline simulation) was random and complex. However, comparatively, the groundwater level decreased under almost all simulation scenarios, while in Big Akaki, predominantly in the upstream area, the level showed a slight increase. The central reason is the distribution of the groundwater pumping wells. The results revealed that the response of the aquifers to the LULC and pumping from upstream to downstream is highly variable. The groundwater head at the upstream area was more sensitive to LULC changes than groundwater abstraction.

**Figure 11:**
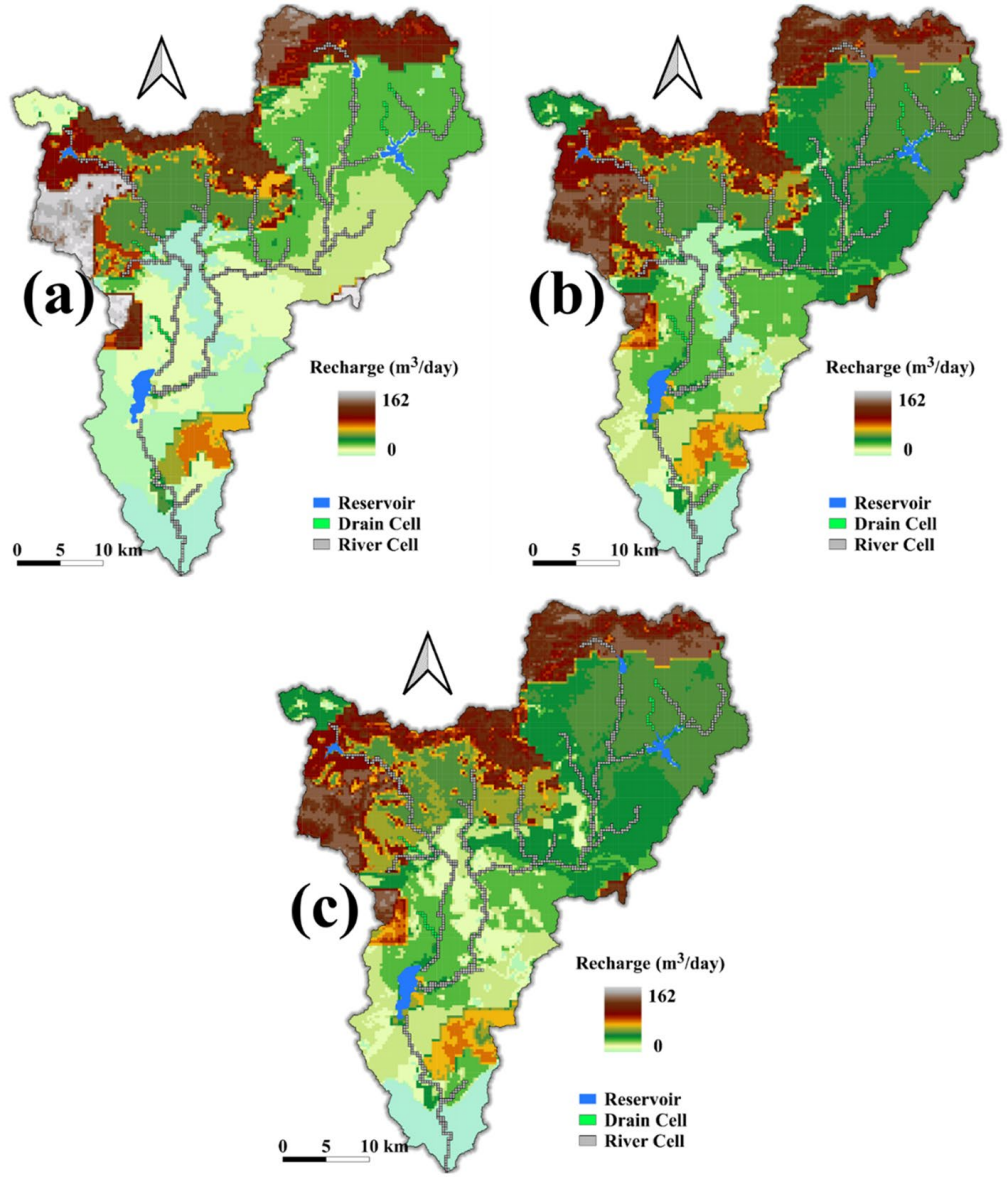
Spatial distribution of average groundwater recharge under baseline (**a**), scenario 2 (**b**), and scenario 4 (**c**) in the study area from SWAT-MODFLOW^44^ output prepared using QSWATMOD2 (https://swat.tamu.edu/software/swat-modflow/).

The most important flow parameter, which affects groundwater level fluctuation and other water balance components, is groundwater recharge. In addition to the results presented in the previous section, the spatial variability of the groundwater recharge related to the LULC change was analyzed. In the region, the groundwater recharge reached a maximum of 162 m^3^/day, and it varied largely with the LULC change (Fig. 11). Surprisingly, as urban expansion increased in the period from 2000 to 2010, the area, which gets moderate to high groundwater recharge, mainly around the boundary of the city, enlarged (compare Fig. 11a, b). The principal reason could be the slight increase in Forest and Shrubland coverage in that period. Compared to scenario 2, the average recharge under scenario 4, particularly in the urban area, increased slightly (Fig. 11c).

### Nitrate mass seeping into the aquifer

**Figure 12:**
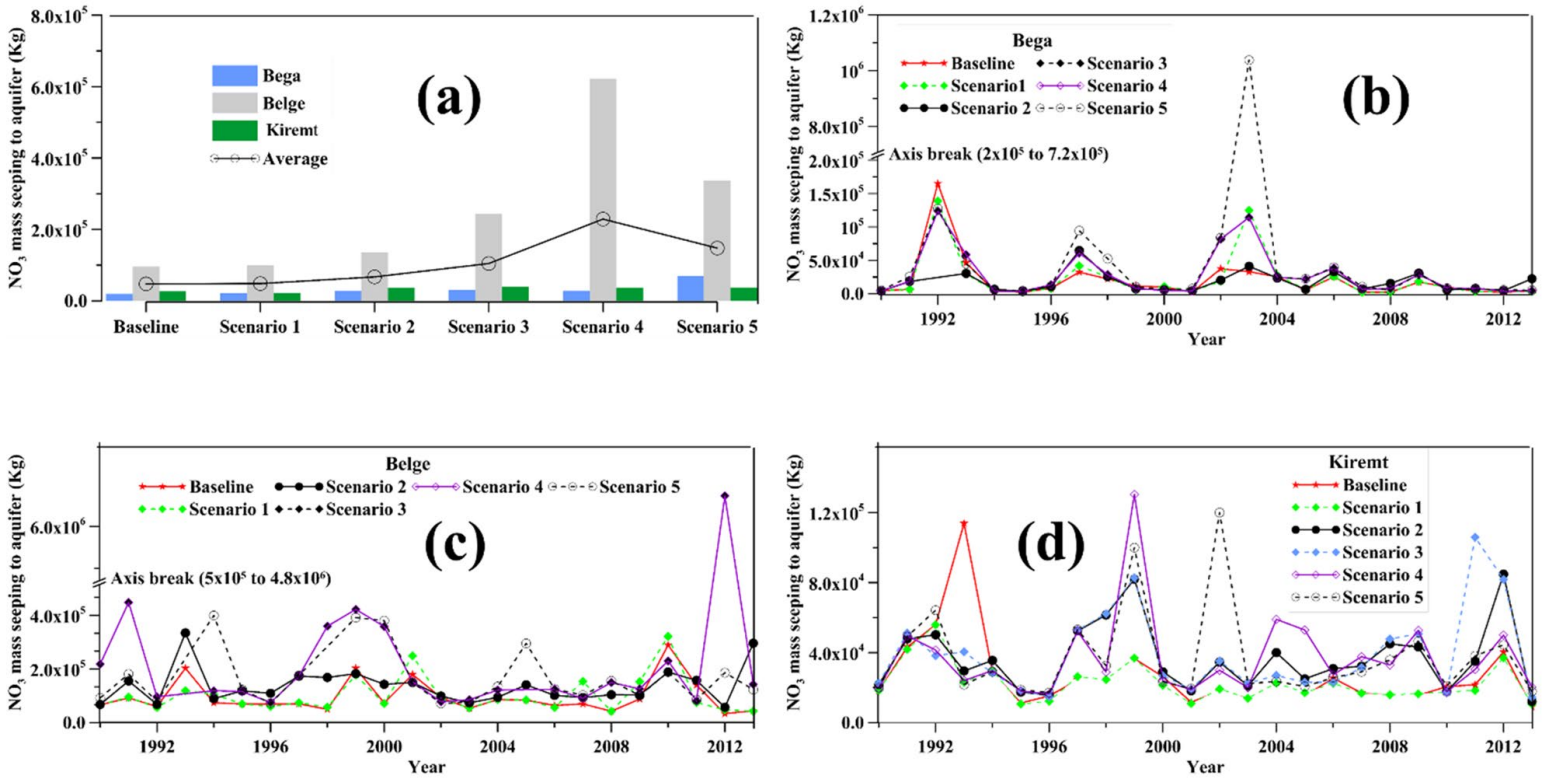
Temporal distribution of average nitrate (No3) seepage to aquifer: (**a**) comparison of average seasonal No3 seepage in each simulation scenario, (**b**–**d**) Seasonal No3 seepage from 1990 to 2013 under all simulation scenarios.

**Figure 13:**
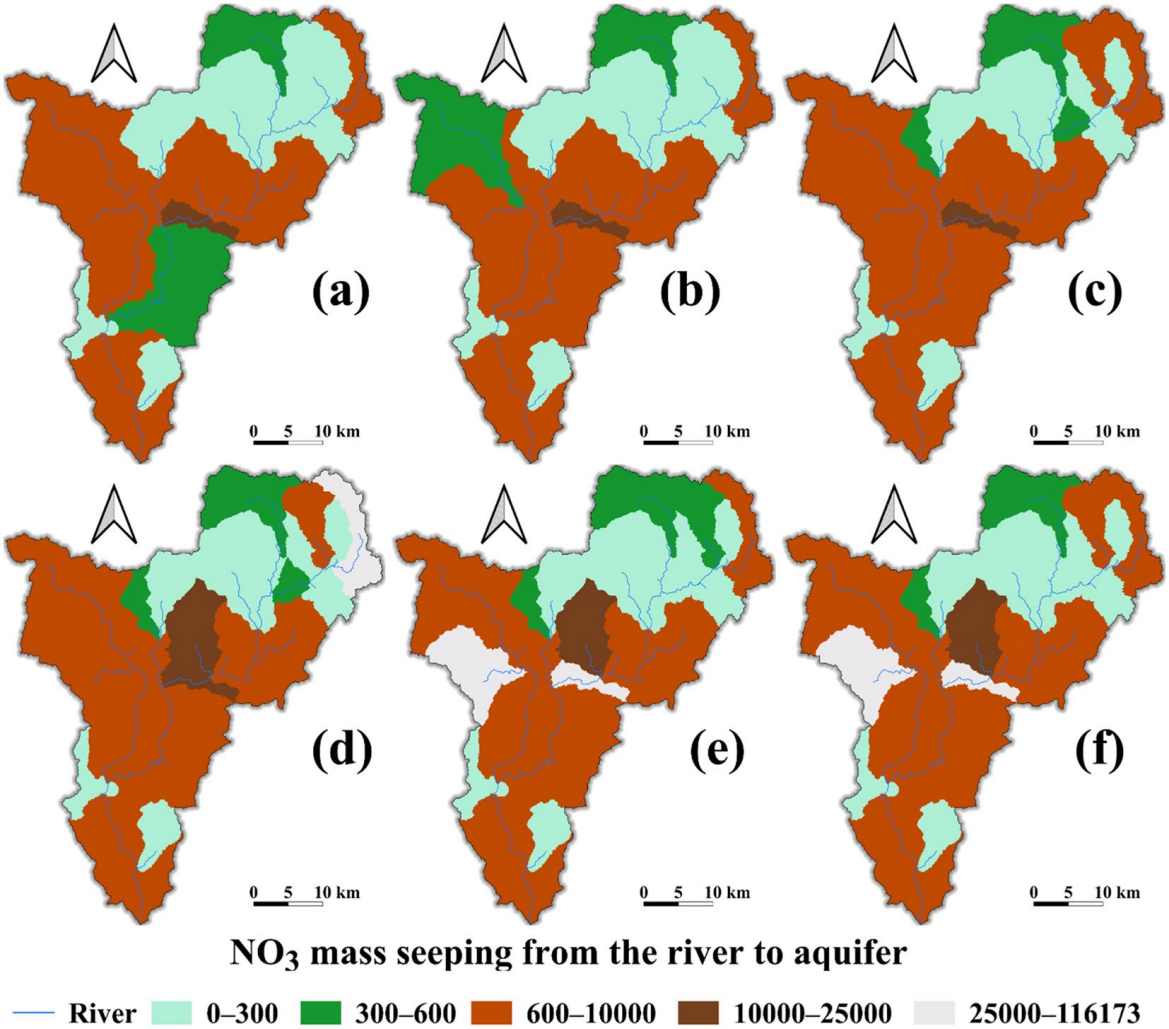
Spatial distribution of nitrate (NO3) seepage (Kg/day) from the river to the aquifer: (**a**) baseline, (**b**) scenario 1, (**c**) scenario 2, (**d**) scenario 3, (**e**) Scenario 4, and (**f**) Scenario 5.

The effect of urbanization on the average spatiotemporal distribution of nitrate mass seepage from the river to the aquifer is presented in Figs. 12 and 13. The seasonal distribution shows that scenarios 4 and 5 increased the nitrate seepage significantly (Fig. 12a). The seepage was high in the Belge under all simulation scenarios comparatively. These results reasonably mirror the finding on water seepage from the river to the aquifer. However, the peaks and lows trend varied from year to year over the simulation period (Fig. 12b–d). The potential source for this random nutrient seepage over the years could be the groundwater recharge change corresponding to the LULC changes.

The average spatial distribution of nitrate mass seepage, evaluated on the SWAT model subbasin scale, revealed that the seepage was moderate in a large part of the region (600–10,000 kg/day). In most of the areas, the average nitrate seepage increased up to 17–250% from the baseline attributed to the urban expansion. However, in scenario 1, the seepage declined by about 4%. This shows that the average nitrate seepage in the region is more sensitive to LULC change than groundwater pumping. The groundwater pumping effect was significant around Aba Samuel lake, also known as the Akaki well-field (Fig. 13a, b). But the effect had no distinct relationship with the spatial distribution of nitrate seepage in all other scenarios (Fig. 13c–f). A maximum of 116,173 kg/day mass of nitrate seeps into the aquifer (averaged over the simulation period). Most of the area covering high seepage was in the Little Akaki, the most urbanized subbasin of the region (Fig. 13e, f).

## Summary and conclusions

In this work, the effect of urbanization on SGI and nitrate loading was assessed using an integrated SWAT-MODFLOW-RT3D model. The changes in SGI and nitrate seepage related to the LULC change and groundwater pumping were evaluated related to the baseline simulation results. In the simulation of nitrate transport, we have incorporated both point and non-point contaminant sources.

The decadal LULC changes analysis showed that the urban area expanded rapidly in the recent decade. Especially, grassland and artificial surfaces had a continuous change. The water balance was more sensitive to LULC changes compared to groundwater abstraction. Among the principal water balance components, the effect of LULC on later flow was substantial. The annual average water seepage from the river to the aquifer decreased by over 4%, while the groundwater discharge decreased by less than 2%. The water yield decreased up to 3% under groundwater abstraction scenarios and up to 9% in LULC change scenarios. Significant changes were found in the Belge season. The combined urban expansion and groundwater pumping compounded the water balance and nitrate seepage changes. The relation between water and nitrate seepage from the river to the aquifer did not exhibit any unique relationship. High nitrate loading was found attributed to LULC changes, and the effect of groundwater abstraction was small. On average, nitrate seepage increased by 17–250% from the baseline in most of the area.

This overarching modeling work highlighted the effect of urbanization usually accompanied by an expansion of the built area, groundwater over-abstraction, and surface water pollution, principally in developing countries with poor waste management on groundwater. Even though the focus was not on providing calibrated and validated model, this work relied on limited data, and the results must be interpreted with caution. Incorporating detailed and longtime-recorded data, different contaminant types, and simulation scenarios are suggested for future studies. Further investigation on aquifer recovery and protection strategies is needed for sustainable water resource management in the region.

## Data Availability

The datasets that support the findings of this study are available from the corresponding author on reasonable request.
